# Recurrent Prenatal‐Postpartum Stress Mediates Acute Cognitive Dysfunction and Alzheimer’s Disease‐Relevant Pathology in Primiparous Rats

**DOI:** 10.1002/alz.084969

**Published:** 2025-01-03

**Authors:** Edem E Edem, Stress and Neuroimmunology Group

**Affiliations:** ^1^ Afe Babalola University, Ado‐Ekiti, Ekiti Nigeria

## Abstract

**Background:**

Stress during pregnancy and postpartum periods has been associated with short‐term cognitive deficits with potential long‐term Alzheimer’s disease (AD) risk. However, the biological mechanisms mediating these effects remain poorly understood. This study investigated the impacts of recurrent heat and simulated refugee camp stress across pregnancy and the postpartum period on cognition, affective behaviour, and AD neuropathological changes in primiparous rats.

**Methods:**

Control dams underwent normal housing while stress dams were exposed to daily prenatal heat (38°C) from gestational day 12‐20 alternating with overnight food/water deprivation and overcrowded conditions to model refugee crises. This recurrent stress continued weekly from postnatal days 23‐37 (postweaning of the first litters). Learning and memory were assessed using the Y‐maze and V‐maze novel object recognition (NOR) tests. Anxiety‐ and depressive‐like behaviours were determined using the elevated plus maze (EPM) and sucrose splash tests, respectively. Following behavioural analyses, hippocampal and frontal cortical levels of pathogenic AD biomarkers amyloid‐beta 42 (Aβ42) and phosphorylated tau (p‐tau) were measured along with neuroinflammation and synaptic integrity markers.

**Result:**

Recurrent prenatal‐postpartum stress induced acute cognitive impairment including impaired recognition memory and spatial learning and memory, increased anxiety‐like behaviour, and mediated AD‐relevant pathological changes including elevated hippocampal and cortical amyloid‐beta aggregation, tau hyperphosphorylation, neuroinflammation, and synaptic alterations compared to the unstressed control group.

**Conclusion:**

Recurrent prenatal‐postpartum stress elicits both immediate neurobehavioral dysfunction and pathological changes potentially increasing AD susceptibility in primiparous rats. These findings may inform interventions to mitigate recurrent perinatal stress impacts on maternal cognitive function and resilience against neurodegeneration.

